# Returning to school: A guide for nurses

**DOI:** 10.1097/01.NURSE.0000942824.50155.a8

**Published:** 2023-07-20

**Authors:** Tracy Jones-Darnell

**Affiliations:** **Tracy** **Jones-Darnell** is an assistant professor of nursing at Nightingale College in Salt Lake City, Utah.

The COVID-19 pandemic has prompted nurses to reflect on better working conditions and work-life balance. The 2022 ACCN annual survey found that applications in all degree levels, except a research-focused doctorate, have decreased from the previous year, potentially validating industry leaders' concerns that the pandemic would discourage enrollment in nursing programs.^1^ This is alarming news because fewer medication errors, lower mortality, and quality patient outcomes are all linked to having nurses prepared at a baccalaureate or higher degree level.^2^

Even prior to the pandemic, a 2019 survey found that nurses were choosing to leave the bedside due to aspects such as demanding schedules, rigid practice settings, and lack of support from management.^3^ Given the multitude of issues in the nursing profession, some nurses are considering going back to school to advance their education to escape the bedside. Similarly, others regard bedside nursing as a stepping stone to career advancement. Increased salary, more manageable work hours, workload, and job satisfaction are some of the alluring opportunities offered by an advanced degree.^3^

## Pre-degree considerations

When considering a school and program, it is important to understand what one can gain professionally and personally. If choosing to advance one's degree, there are numerous routes to consider. Bachelor of Science in Nursing (BSN) and Master of Science in Nursing (MSN) are the most popular degrees conferred for nurses, but many nurses do not know the different academic requirements for the degrees nor how each degree will help accomplish their career goals.^4^

When searching through various school and degree options and trying to decipher the unfamiliar language of school catalogs, students may feel these are unachievable time-consuming tasks to accomplish. Cadet^5^ reported that when nursing students are not prepared for online learning in particular, attrition rates increase. To combat this issue, students need to consider components such as a plan of study, student services, and clinical site placements and review each school's educational options (in-person, online, or a hybrid) to determine which will best fit their lifestyle. Additionally, work obligations and scheduling need to be considered to ensure students' learning environment focuses on learning and not task completion.

## Getting started

Learning is an integral part of obtaining a degree, but it is often the most overlooked component.^6^ The ability and readiness to learn are critical components of school success. The ability to learn is demonstrated in nurses' successful completion of rigorous prelicensure programs to become an RN. However, and more important, nurses must assess if their readiness to learn is present before and above all else.

To be ready to learn, one must consider the time and financial commitment required to attend school. Interestingly, Cadorin^6^ reports that self-directed learners will be more successful as they overcome the challenges in nursing school with ease. Diab and Elgahsh^7^ reported that the biggest obstacles for students, identified in the first year of their program, were all based on the support of the school and instructors.

What is meant by time commitment? A student needs to secure family and employer support so that the necessary time can be devoted to school without additional barriers. Family members will have to allow time for schoolwork without household distractions. Employers will have to allow time off when clinical or classroom assignments are due.

## Financing options

Financial commitment is often the determining factor for going back to school (see *Financing options*). Some healthcare facilities offer tuition reimbursement for their employees. Nurses considering this option must understand any specific work requirements required after graduation. In some instances, a facility may require the nurse to work for 1 to 2 years after completion. For nurse educators, the college or university could require them to teach for a certain number of years.

Loan and grant options can be accessed directly through a school's financial aid department. Three basic categories of financial aid are available: Gift aid, which are scholarships and grants that do not require repayment; loans; and part-time employment with the federal work-study program.^8^ A student's financial aid packet is generally made up of two or more categories. Students who are obtaining their first bachelor's degree will be considered for all three types of assistance, whereas those pursuing another bachelor's degree are only considered for loans and work assistance.^8^

First, one must consider eligibility for Federal Pell Grants. These are awarded to students based on financial need who have not earned a bachelor's, graduate, or professional degree. The amount awarded is based on financial need, costs to attend school, full-time or part-time status, and a plan to attend school for up to a full academic year.

After determining if one is eligible for Pell Grants, then one can consider a federal loan that will need to be repaid upon graduation. Among the various federal loans, the Public Health Service Act^9^ allows for several student loans for nursing workforce development. Nursing student loans are available for associate, bachelor's, and graduate degrees in nursing. The Nursing Faculty Loan program is available to those pursuing graduate degrees who are interested in becoming a nursing faculty. The program provides a loan cancellation benefit in exchange for working as a nursing faculty for 4 years.

**Table:** Financing options^8^,^9^

Grants or Pell Grant	Focus on a student's financial aid need or merit-based aid Do not need to be repaid Can be used for up to 12 full-time semesters Those who have not earned a bachelor, graduate, or professional degree
Federal student loans	Must be repaid Can be from any qualifying lending agency Amount of loan is dependent on the student
Scholarships	Merit-based and needs-based Do not need to be repaid High school or college board opportunity scholarships Amount varies on the awarded scholarship
State assistance	State-based programs funded by external agencies In Georgia, Hope grant funded by Georgia Lottery Commission
Work-study program	Need-based Earn instead of gifted or borrowed Federal work-study program is an example
Nurse Reinvestment Act	Component of the Public Health Service act Nurse scholarship and loan repayments for those agreeing to work in nursing shortage areas
Nurse Faculty Loan	Available for nurses who are pursuing a career as a nurse faculty Low interest loans Loan cancellation in some instances

Eligibility needs are based on the need of the individual as well as borrowing parameters.^8^ When considering any type of financial aid, the student should borrow with the mindset that the money will have to be repaid.^9^

## Selecting a program

After considering financial and time commitments, one then must choose a program. There are many more program options than there were just a decade ago. These include face-to-face, online, and hybrid programs (a combination of in-person and online learning activities). The BSN, MSN, DNP, and PhD degrees are available in all of these formats and have multiple specialty options for each degree.

The prospective post-licensure student population today is more diverse than a decade ago.^10^ Students from minority racial and ethnic groups make up over a third of students in post-licensure programs; many are students from other countries where English is their second language.^1^ Approximately 11% of all current nursing students are male. Many students are in nursing as their second career. Student demographics vary from program to program and state to state. A prospective student should peruse the school website and verify that the school's mission, vision, and value statements align with their personal values and preferences, including considerations related to diversity, equity, and inclusion.

### Accreditation

An often-overlooked component when considering which school to attend is accreditation–a process that makes all nursing schools adhere to the same quality standards set forth by the United States Department of Education.^11^ The goal of accreditation is to ensure that institutions of higher education meet acceptable levels of quality. Accreditation is important because it ensures that a student in California is getting the same quality of education as someone across the country in Vermont at another nursing school. Accreditation allows the school to provide federal financial aid options to its students as well as transfer previously earned contact hours from other institutions.^8^

Nursing programs are accredited at different levels. Nursing's primary accrediting bodies are the Accreditation Commission for Education in Nursing (ACEN) and the Commission on Collegiate Nursing Education (CCNE). ACEN is recognized by the US Department of Education and the Council for Higher Education Accreditation and is a subsidiary of the National League for Nursing. ACEN accredits all levels of nursing education programs and transition to practice programs in the US and US territories as well as internationally.^11^ CCNE is recognized by the US Secretary of Education as an autonomous arm of the AACN. CCNE accredits bachelor's, master's, and doctoral-level nursing programs, as well as entry-to-practice nurse residency programs and nurse practitioner fellowship and residency programs in the US.^12^

Two other accrediting bodies exist for the specialized graduate programs: the Council on Accreditation of Nurse Anesthesia Educational Programs (COA)^13^ and the American College of nurse Midwives Divison of Accrediation (ACNM)^14^. In addition, COA accredits nurse anesthesia programs, and ACNM accredits midwifery programs.

Silvestre^15^ reported that there is a decrease in accreditation in baccalaureate and graduate programs, which could be attributed to the increase in newer programs. A school's accreditation may not be clear on its application; however, it should be easily located on the school's website.

It is important to be an informed consumer, as many employers will not hire graduates from certain programs with canceled or suspended accreditation status. Additionally, several states have strict guidelines on who can practice. One of the most important criteria considered is graduation from an accredited nursing program.

## Baccalaureate

In 2010, the Institute of Medicine (IOM) challenged the nursing profession in its report, The Future of Nursing, to increase the number of BSN nurses in the workforce.^16^ In 2010, 49% of nurses were BSN-prepared; in 2022, 56% were BSN-prepared.^17^ According to the AACN 2022 annual online survey, there were 747 RN-to-BSN programs and 844 entry-level baccalaureate programs in the US that year.^1^

AACN^18^ reported in 2022 that 28% of employers of hospitals and other healthcare settings are requiring a BSN for their new hires, and 72% of employers report preferential hiring of BSN graduates over diploma or ASN nurses. Dimattio and Spegman^3^ reported a positive relationship between educational preparedness and positive patient outcomes in acute care settings. Baccalaureate nursing education consists of additional coursework in nursing research, leadership and management, and public and community health nursing. This preparation allows employers to view BSN-prepared nurses as future leaders or managers educated in evidence-based practices.^2^

To clearly understand the professional benefits of having a BSN, nurses can take a moment to search online job boards and explore online and in-person jobs available for BSN nurses in roles in insurance, case management, leadership, staffing, and academia. For instance, in academia, a BSN nurse is qualified to teach both certified nursing assistants and LPNs/LVNs. Nurses who enjoy orienting and precepting new staff may want to consider nursing education.

Baccalaureate programs are important pathways for nurses to increase their knowledge base and strengthen their nursing skills to better meet patient care needs. With over 700 programs in the US in 2019, an associate or diploma nurse has several options for obtaining a bachelor's degree.^19^ As of this writing, 781 RN-to-BSN programs are available in the US, of which 650 are offered partially or fully online.^4^ Both program types consist of approximately 1 year of coursework with varying clinical requirements. Students can attend full-time and complete the course work in the minimum time or attend part-time students. Attending part-time is an excellent way to go back to school while working full-time and taking care of family obligations. Part-time coursework has the same requirements as full-time and can be completed at a slower pace. Sometimes students begin as full-time students and realize their obligations will only allow them to go to school part-time. This change is manageable, and one does not have to apply to another program.

## Master's

The 2022 AACN^17^ survey indicated that the number of nursing roles requiring an MSN may grow by 45% by 2030. This will change the landscape of the nursing profession. The number of primary care providers is estimated to decline while the number of NPs is expected to increase, so more patients may see NPs as their primary care providers.^20^

Nurses with an MSN possess a skill set of advanced treatments, broad system-level thinking, and leadership skills that nurses with a BSN do not. These skills may not be directly tied to patient care but are necessary for advancement in the nursing profession. An MSN degree will open doors for additional opportunities in nursing. MSN-prepared nurses can hold leadership positions in the hospital or academia; consult on health policy; conduct research; or provide direct patient care as an APRN in acute, home, or long-term settings.

MSN-prepared nurses have more autonomy than BSN-prepared nurses and, except for those in academia, are typically paid at a higher hourly rate.^21^ As with RNs, the MSN pay scale can fluctuate depending on the geographical location and the area of practice or specialty.^21^ With the expanding interest in the MSN degree, schools are adapting to offer new specializations that allow nurses to develop specific skill sets.^22^ Specialization is beneficial to improving patient outcomes and creates a niche skillset.

The most popular option in MSN programs is the Nurse Practitioner track, but other options include Nurse Educator, Clinical Nurse Specialist, Nurse Executive, Nursing Informaticist, Nurse Anesthetist, and Nurse Midwife.^21^ According to the US Bureau of Labor Statistics,^23^ the median yearly salary for NPs, CRNAs, and nurse midwives is approximately $124, 000. While the CRNA role is reported to have the highest yearly salary, a nurse educator's yearly salary is reported to be the lowest at approximately $78,000 per year.^23^

Work-life balance must also be considered when pursing an MSN. Most MSN programs require a significant number of clinical hours in addition to coursework, with as many as 1,300 total clinical hours.^24^ Some programs require MSN students to complete projects before graduation and occur in the last semester, whereas other programs require a written, research-based thesis that can take over a year to complete after the coursework completion.

As of 2022, there were 659 Master's programs in the US. Much like the BSN programs, MSN programs vary widely according to the track and specialty. There are face-to-face, online, and hybrid formats, with 198 of those being RN-to-MSN bridge programs for ADN or diploma nurses.^4^ Most BSN-to-MSN programs take 2 years with the completion of a thesis or project; the typical RN-to-MSN bridge program takes 3 years, with the first year of bridge programs consisting of mastering the upper-level basic nursing content. As with a BSN, part-time enrollment is available to students who have work and family commitments that may prohibit them from attending school full-time.

## Doctorate

Two doctoral degrees, a Doctor of Nursing Practice (DNP) and a Doctor of Philosophy (PhD), are available for nurses. When deciding between the different doctoral degrees, it is important to first determine the overall professional goal and what professional role it will be used for after graduation.

Both the DNP and PhD are considered terminal nursing degrees.^25^ In general terms, a DNP is a clinical practice degree, and a PhD is a research-focused degree. If one's interests in nursing are to discover, research, and disseminate new nursing knowledge, then a PhD in Nursing is the best option. If one is more interested in translating knowledge gained from nursing research into practice and improving systems of care for patient groups, or communities, then a DNP degree may be more appropriate.^26^ While this sounds like a simple difference, it is the only similarity between the two degrees. The DNP degree allows practice experts to take the knowledge from nursing research, review the evidence, evaluate its impact on nursing practice, and make the necessary changes to enhance the quality of care offered to their patients.^26^

Although PhD- and DNP-prepared nurses have different educational preparedness and skill sets, it is vital that they collaborate to improve the nursing profession and help educate future nurses.

### DNP

The DNP degree was originally created for APRNs, including nurse midwives, CRNAs, and clinical nurse specialists to become leaders in clinical practice. This is similar to pharmacy and physical therapy who moved their disciplines to a practice doctorate.^26^

Nursing leaders with practice doctorates are needed to deliver care, determine healthcare policy, develop healthcare standards, and assist in reducing disparities in healthcare accessibility and delivery through the anticipated nursing shortage.^27^ In 2004, the AACN endorsed the DNP as the most appropriate degree for entry-level advanced practiced nurses, with a goal to have all APRNs obtain a DNP by 2015.^26^ The IOM did not address the adoption in their 2010 report.^16^

Although the AACN's goal goal was not met by 2015, DNP programs have seen more than 20 years of continuous growth and have 10 times the number of enrolled students than PhD programs.^26^ The growth is partly due to programs admitting students with MSN degrees other than APRNs and focusing on developing leadership initiatives versus clinical proficiency.^26^

Most DNP programs are based on 1 to 2 years of online coursework but can extend to 4 years if taken as a part-time student. There are typically 30-40 course contact hours with approximately 1,000 clinical hours depending on the school.^25^ A project or capstone project is typically required for graduation and is completed in the last semester of the program.

While the DNP degree did not develop a stronger presence in primary care settings, it helped contain the shortage of nursing faculty. and increase the proportion of doctoral-prepared nursing faculty. The issue nursing programs have today is how to concurrently educate experienced NPs and post-BSN students who have little to no clinical experience. Maintaining consistency within DNP programs is challenging for schools attempting to provide a consistent DNP curriculum given the wide variety of student clinical experiences.

Given the stringent PhD requirements that may be unattainable for some students, the DNP provides an option that will produce additional practice experts, although it does create difficulties in improving the healthcare community's understanding of a DNP-prepared nurse's competencies.^26^

Lastly, degree options are based on a state-by-state case. Some states, like New York, do not provide DNP options for nurses who are not associated with an NP, midwife, or CRNA, while other states like Georgia provide an array of options from clinical tracks to leadership and education tracks.^28^ State practice laws for NPs are also important as some states grant full-practice authority, while others have limited practice authority. The state laws are fluid, and advancements are made every year in NP practice laws. It is important to keep abreast of the changes.

### PhD

The IOM^16^ advocated for the doubling of PhD-prepared nurses by 2020. However, the US fell short of this recommendation: 1% of the nursing workforce has earned a PhD.^29^ Furthermore, enrollment in PhD programs declined over the last 10 years and continues to decline. Puzantian and Darwish^30^ suggest that the future of nursing depends on nursing scholars who investigate and generate new knowledge for nursing science. Doctorally prepared faculty are in high demand to ensure nursing has a presence in health science research and the development of evidence-based practices.^31^ The PhD degree has been the preferred degree for academic nursing faculty due to the preparation of research and scientific study.^31^

Factors that may be associated with students' lack of interest in pursuing a PhD include assumed unachievable goals, the programs' rigor, admission criteria, and graduate record exam (GRE) requirements. However, the greatest factor that discourages students is the time commitment. The average full-time PhD student is in school for a minimum of 3 years with course work and dissertation requirements. Part-time students usually complete a PhD in 5 to 7 years.^25^ The dropout rate for PhD students is higher than in other programs, which is not surprising as it is difficult to juggle family and work. Given the additional financial and time burden of school, some just give up.^32^,^33^

The timing of a PhD program is based on the program design and dissertation requirements. Most face-to-face programs require 2 years of coursework (theory, research, and experimentation) and then dissertation work The dissertation process involves selecting a topic of interest, compiling research on the topic, and performing a research study to be defended with a presentation and publication. This phase, referred to as the “All But Dissertation” (ABD) phase, can range from 1 year to 5 or more.^29^ The ABD phase is one of the variables found to cause some students to drop out of their PhD program, particularly due to the amount of time and funding needed to solely dedicate to the research study and writing process.^33^ Many cite ABD in their credentials to indicate the completion of school but may end up never completing the requirements for their PhD.

## Conclusion

The nursing profession is rooted in lifelong learning; however, not all nurses need to leave the bedside to pursue an advanced degree. The healthcare system is dependent on clinical nurses caring for the injured and sick. Regardless of what degree is pursued, future scholars, research scientists, and educators bring promise to the nursing profession.

## References

[1] AACN. Highlights from AACNs 2021 Annual Survey. 2022. https://www.aacnnursing.org/Portals/42/Publications/Annual-Reports/2022-AACN-Annual-Report.pdf

[2] Park SH Gass S Boyle DK. Comparison of reasons for nurse turnover in Magnet^®^ and non-Magnet hospitals. *J Nurs Adm*. 2016;46(5):284–290. doi:10.1097/NNA.0000000000000344. 27093185 10.1097/NNA.0000000000000344

[3] DiMattio MJK Spegman AM. Educational preparation and nurse turnover intention from the hospital bedside. *Online J Issues Nurs*. 2019;24(2).

[4] AACN. Degree completion programs for registered nurses: RN to master's degree and RN to baccalaureate programs. 2022. www.aacnnursing.org/Portals/42/News/Factsheets/Degree-Completion-Factsheet.pdf.

[5] Cadet MJ. Examining the learning characteristics of nursing students: a literature review. *J Nurs Educ*. 2021;60(4):209–215. 34038279 10.3928/01484834-20210322-05

[6] Cadorin L Bressan V Palese A. Instruments evaluating the self-directed learning abilities among nursing students and nurses: a systematic review of psychometric properties. *BMC Med Educ*. 2017;17(1):229. doi:10.1186/s12909-017-1072-3. 29178924 10.1186/s12909-017-1072-3 PMC5702155

[7] Mohamed Abd El-Hamed Diab G Fouad Elgahsh N. E-learning during COVID-19 pandemic: obstacles faced nursing students and its effect on their attitudes while applying it. *Am J Nurs Sci*. 2020;9(4):300. doi:10.11648/j.ajns.20200904.33.

[8] Federal Pell Grant Program. An Office of the U.S. Department of Education. 2023. https://www2.ed.gov/programs/fpg/index.html.

[9] Heisler EJ Hegji A. Student Loan Programs Authorized by the Public Health Service Act: An Overview. CRS Report R46720, Version 1. *Congressional Research Service*. 2021.

[10] National Academies of Sciences, Engineering, and Medicine; National Academy of Medicine; Committee on the Future of Nursing 2020-2030; Flaubert JL, Le Menestrel S, Williams DR, et al., editors. The Future of Nursing 2020-2030: Charting a Path to Achieve Health Equity. Washington (DC): National Academies Press (US); 2021 May 11. 3, The Nursing Workforce. https://www.ncbi.nlm.nih.gov/books/NBK573922/34524769

[11] Accreditation Commission for Education in Nursing (ACEN). ACEN website. 2020. www.acenursing.org/about/recognition/.

[12] Commission on Collegiate Nursing Education (CCNE). What we do. American Association of Colleges of Nursing. 2023. https://aacnnursing.org/CCNE-Accreditation/What-We-Do.

[13] Council on Accreditation of Nurse Anesthesia Educational Programs (COA). Accreditation. 2020. www.coacrna.org/.

[14] Accreditation Commission for Midwifery Education (ACME). 2023. www.midwife.org/acme.

[15] Silvestre JH. Percentages of programs that are accredited: an update. *Leader to Leader*. 2020. https://ncsbn.org/public-files/Percentages_Accredited_Programs_L2L.pdf.

[16] Institute of Medicine. *The Future of Nursing: Leading Change, Advancing Health*. Washington, DC: National Academies Press; 2010. 24983041

[17] AACN. Fact Sheet: the impact of education on nursing practice. 2022. www.aacnnursing.org/news-information/fact-sheets/impact-of-education.

[18] AACN. Employment of new nurse graduates and employer preferences for baccalaureate prepared nurses. 2022. www.aacnnursing.org/Portals/42/News/Surveys-Data/Research-Brief-10-21.pdf.

[19] AACN. Nursing Fact Sheet. 2022. www.aacnnursing.org/News-Information/Fact-Sheets/Nursing-Fact-Sheet.

[20] Bauer L Bodenheimer T. Expanded roles of registered nurses in primary care delivery of the future. *Nurs Outlook*. 2017;65(5):624–632. doi:10.1016/j.outlook.2017.03.011. 28483137 10.1016/j.outlook.2017.03.011

[21] Bureau of Labor Statistics, U.S. Department of Labor. Occupational outlook handbook: registered nurses. 2018. www.bls.gov/ooh/healthcare/registered-nurses.htm#tab-3.

[22] Dierkes AM Schlak AE French R McHugh MD Aiken L. Why some nurses obtain specialty certification and others do not. *J Nurs Adm*. 2021;51(5):249–256. doi:10.1097/NNA.0000000000001009. 33882552 10.1097/NNA.0000000000001009 PMC8064627

[23] Bureau of Labor Statistics, U.S. Department of Labor. Occupational Outlook Handbook, Nurse Anesthetists, Nurse Midwives, and Nurse Practitioners. 2021. www.bls.gov/ooh/healthcare/nurse-anesthetists-nurse-midwives-and-nurse.

[24] NurseJournal.org. Getting clinical experiences as an online nursing student. https://nursejournal.org/resources/clinical-experience-for-online-nursing-students/.

[25] Kathleen Gaines. DNP vs Ph.D. in Nursing - what is the difference? 2019. https://nurse.org/education/dnp-or-phd-in-nursing-difference/.

[26] McCauley LA Broome ME Frazier L Doctor of nursing practice (DNP) degree in the United States: reflecting, readjusting, and getting back on track. *Nurs Outlook*. 2020;68(4):494–503. doi:10.1016/j.outlook.2020.03.008. 32561157 10.1016/j.outlook.2020.03.008 PMC7161484

[27] Juraschek SP Zhang X Ranganathan V Lin VW. United States registered nurse workforce report card and shortage forecast. *Am J Med Qual*. 2019;34(5):473–481. doi:10.1177/1062860619873217. 31479295 10.1177/1062860619873217

[28] Doctor of Nursing Practice DNP. A state-by-state guide to online and campus-based CCNE and ACEN-accredited DNP programs. www.doctorofnursingpracticednp.org/accredited-dnp-programs-by-state/.

[29] American Association of Colleges of Nursing. Data Spotlight: trends in nursing PhD programs. 2020. www.aacnnursing.org/news-data/all-news/article/data-spotlight-trends-in-nursing-phd-programs.

[30] Puzantian H Darwish H. Redesigning a PhD measurement course for a new era in nursing science. *J Prof Nurs*. 2021;37(2):387–390. doi:10.1016/j.profnurs.2020.04.019. 33867095 10.1016/j.profnurs.2020.04.019 PMC7191290

[31] Squires A Kovner C Faridaben F Chyun D. Assessing nursing student intent for PHD study. *Nurse Educ Today*. 2014;34(11):1405–1410. doi:10.1016/j.nedt.2013.09.004. 24080267 10.1016/j.nedt.2013.09.004

[32] Maddox S. Did Not Finish: Doctoral Attrition in Higher Education and Student Affairs. Published Dissertation. 2017. https://digscholarship.unco.edu/cgi/viewcontent.cgi?article=1434&context=dissertations.

[33] Wollast R Boudrenghien G Van der Linden N Who are the doctoral students who drop out? Factors associated with the rate of doctoral degree completion in universities. *Int J High Educ*. 2018;7(4):143–156. https://files.eric.ed.gov/fulltext/EJ1188721.pdf.

